# Full Hematocrit–Viscosity Curve Identification Using Three-Dataset Krieger–Dougherty Regression

**DOI:** 10.3390/bios16040216

**Published:** 2026-04-10

**Authors:** Yang Jun Kang

**Affiliations:** Department of Mechanical Engineering, Chosun University, 10, Chosundae 1-gil, Dong-gu, Gwangju 61452, Republic of Korea; yjkang2011@chosun.ac.kr; Tel.: +82-62-230-7052; Fax: +82-62-230-7055

**Keywords:** K-D regression model, full hematocrit–viscosity curve, three hematocrit–viscosity datasets, blood viscosity, hematocrit, coflowing streams method, micro-hemocytometer, blood separation, microfluidic chip

## Abstract

Blood viscosity is strongly dependent on hematocrit, and the hematocrit–viscosity relationship is an important determinant of blood rheology under physiological and pathological conditions. However, obtaining a full hematocrit–viscosity curve requires multiple measurements over a wide hematocrit range. In this study, a simple method is proposed to reconstruct the full hematocrit–viscosity curve using only three-dataset Krieger–Dougherty (K–D) regression as $\mu ={\mu_{0}(1-\frac{\phi }{\phi_{m}})}^{-\alpha \;\phi_{m}}$. Based on suspended blood, RBC-rich blood and RBC-depleted blood are prepared after centrifugation. The hematocrit of each type of blood is measured using a micro-hemocytometer. Simultaneously, the blood viscosity of each type of blood is measured using the coflowing streams method. The proposed method is evaluated sequentially using reference datasets and hematocrit–viscosity datasets of control blood. According to results, the full hematocrit–viscosity curve obtained from three selected datasets is in good agreement with the experimental data and yields a lower root-mean-square error than conventional methods using all datasets. The exponent of the K–D model is strongly influenced by the midpoint dataset, whereas *μ*_0_ is mainly affected by the suspending medium (dextran solution). In contrast, GA-induced rigidified RBCs do not significantly affect *μ*_0_ within a 0.15% concentration. In conclusion, the proposed method provides a simple, efficient, and reliable approach for estimating the full hematocrit–viscosity curve.

## 1. Introduction

Blood consists of plasma and cellular components (i.e., red blood cells [RBCs], white blood cells, and platelets) [1]. Blood viscosity is a rheological property that represents the resistance of shearing blood flow, which is defined as the ratio of shear stress to shear rate. Clinically, blood viscosity plays a critical role in determining vascular resistance, microcirculatory blood flow, and tissue oxygen supply [2]. An abnormal increase in blood viscosity can hinder blood flow and is closely related to cardiovascular risk [3,4], thrombosis, ischemia, and diabetes [5,6]. Accordingly, blood viscosity is regarded not only as a basic physical parameter of blood circulation, but also as a promising biomarker for evaluating hemorheological abnormalities and the progression of disease.

Blood viscosity is determined by several factors, including, hematocrit [7,8,9,10,11,12,13,14,15], plasma composition [4,16,17,18,19,20,21], and RBC rheological properties (i.e., aggregation [22,23,24,25,26,27,28,29,30] and deformability [31,32,33,34,35,36]). Blood viscosity is governed by the shear rate of blood flow (i.e., non-Newtonian fluid). Among the factors influencing blood viscosity, hematocrit is the most important factor affecting blood viscosity. In other words, when blood hematocrit increases, the RBCs become more crowded, which makes blood flow more difficult. Consequently, it contributes to increasing blood viscosity [1,2,10,15,37,38,39,40,41].

When blood viscosity is described solely as a function of hematocrit, the corresponding regression model has been progressively refined from a simple linear approximation to a nonlinear asymptotic form. The earliest expression is the Einstein-type relation (i.e., $\frac{\mu }{\mu_{0}}=1+2.5\;\phi$). Herein, *μ*, *μ*_0_, and *ϕ* denote blood viscosity, plasma viscosity, and hematocrit, respectively. However, this equation is valid only at very low hematocrit levels (i.e., *ϕ* < 0.1) and does not adequately describe whole blood over the physiological hematocrit range. This limitation is later addressed by the finite concentration model [42] and more fully resolved by the Krieger–Dougherty (K-D) model [43,44], $\frac{\mu }{\mu_{0}}={\left(1-\frac{\phi }{\phi_{m}}\right)}^{-\alpha \;\phi_{m}}$. Herein, a maximum packing factor ($\phi_{m}$) is introduced to represent the steep rise in blood viscosity as hematocrit approaches its upper limit. The exponent (*α*$\phi_{m}$) represents both contributions of RBCs to blood viscosity and maximum hematocrit. Accordingly, the hematocrit–viscosity relation has often been represented by a Krieger-type asymptotic equation [10,45], $\mu =\mu_{0}{\left(1-\frac{\phi }{\phi_{m}}\right)}^{-n}$. These forms describe the hematocrit–viscosity relationship over the full hematocrit range.

The major limitation in blood sample preparation is that the hematocrit intended from volumetric mixing is often different from the hematocrit obtained by a micro-hemocytometer. Considering that hematocrit strongly influences the viscosity of blood suspensions, hematocrit is commonly adjusted by centrifuging whole blood and recombining the concentrated red blood cell fraction with plasma or another suspending medium to obtain nominal target values [10]. However, the packed red blood cell layer obtained after centrifugation is not a pure cell phase because a small amount of plasma is trapped within the packed layer [46,47]. In addition, manual microhematocrit determination introduces reader-dependent variability and other measurement errors, which further complicate the accurate adjustment of hematocrit [48]. Therefore, when blood samples are prepared by pipetting fixed volumes of concentrated red blood cells and medium, the initially intended hematocrit may differ from the measured value, making post-preparation verification and repeated readjustment essential for obtaining a precise target hematocrit. This is especially important in viscosity experiments, where even small hematocrit deviations can alter the measured hematocrit–viscosity relationship [49]. To minimize hematocrit mismatch during sample preparation, the packed RBC fraction is washed two or three times to reduce residual plasma. The prepared suspension is verified using a standardized hematocrit method, and the mixing ratio is then readjusted on the basis of the measured hematocrit rather than the nominal volumetric value [13,14,50]. More recently, hematocrit has been measured by detecting the interface position in a capillary channel [9,51].

In this study, a new method is proposed to identify the full hematocrit–viscosity curve from three hematocrit–viscosity datasets based on the K–D regression model. First, the hematocrit of suspended blood is measured using a centrifuge hemocytometer and is denoted as *ϕ*_1_. Its corresponding viscosity (*μ*_1_) is measured by flowing the sample through a microfluidic chip. Next, centrifugation of the driving syringe separates the blood into RBC-rich and RBC-depleted layers. The hematocrit of the RBC-rich layer is measured as (*ϕ*_2_), while the viscosities of the RBC-rich and RBC-depleted layers are obtained as *μ*_2_ and *μ*_3_, respectively. The hematocrit of the RBC-depleted layer is assumed to be *ϕ*_3_ = 0. Finally, based on the K–D regression model as $\mu ={\mu_{0}(1-\frac{\phi }{\phi_{m}})}^{-\alpha \;\phi_{m}}$, the full hematocrit–viscosity curve is determined from the three hematocrit–viscosity datasets (i.e., *ϕ*_1_-*μ*_1_, *ϕ*_2_*-μ*_2_, and *ϕ*_3_-*μ*_3_).

Compared with the conventional method that requires multiple separately prepared blood samples at different hematocrit levels, the proposed method offers several advantages. First, it reduces repeated hematocrit adjustment by reconstructing the full hematocrit–viscosity curve from only three datasets. Second, it simplifies the experimental procedure by generating RBC-rich and RBC-depleted fractions directly from a single sample. Third, it decreases blood consumption, preparation time, and inter-sample variability, thereby providing a more efficient and consistent approach for identifying the full hematocrit–viscosity relationship.

## 2. Materials and Methods

### 2.1. Measurement of Hematocrit and Blood Viscosity

To obtain full hematocrit–viscosity curve, it was necessary to get hematocrit and blood viscosity, respectively.

**Figure 1:**
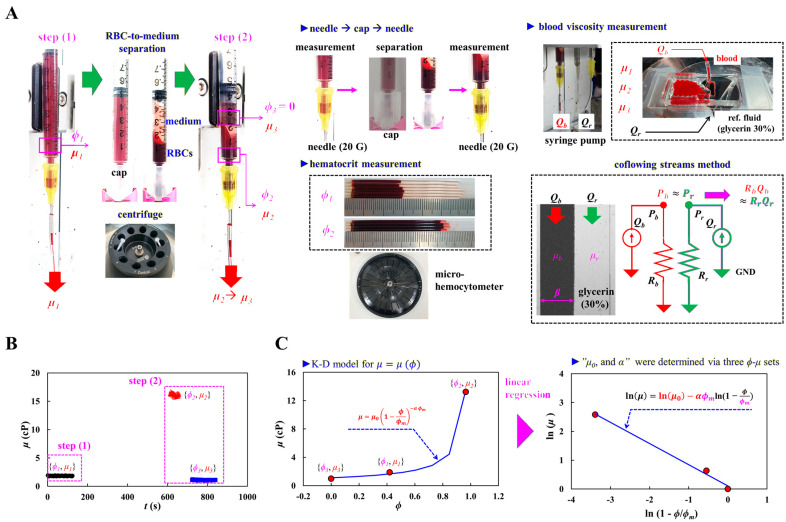
A proposed method for obtaining the full hematocrit–viscosity curve. (**A**) Experimental setup including the micro-hemocytometer for hematocrit (*ϕ*) and a microfluidic system for blood viscosity (*μ*). The left-side panel shows a two-step measurement of hematocrit and viscosity. The middle-side panel shows replacement of a syringe needle for RBC-to-medium separation and a micro-hemocytometer for hematocrit measurement. The right-side panel shows blood viscosity measurement using the coflowing streams method. (**B**) Time-lapse blood viscosity was obtained through two steps of measurement. (**C**) Calculation procedure of full hematocrit–viscosity curve using K-D regression model. The left-side panel shows the full hematocrit–viscosity curve using the K-D model: $\mu =\mu_{0}{\left(1-\frac{\phi }{\phi_{m}}\right)}^{-\alpha \phi_{m}}$. Three hematocrit–viscosity datasets are plotted on the hematocrit (*ϕ*) and viscosity (*μ*) axes. The right-side panel shows linear regression analysis for obtaining two unknown variables (i.e., *μ*_0_ and α), where the *ϕ_m_* is specified as *ϕ_m_* = 1. The regression analysis estimated two unknown constants.

As shown in Figure 1A, an experimental setup was composed of a micro-hemocytometer for hematocrit (*ϕ*) and a microfluidic system for blood viscosity (*μ*). As shown in the left-side panel, procedures for measuring hematocrit and blood viscosity were divided into two steps. At the first step, with regard to suspended blood, hematocrit of suspended blood was obtained as *ϕ*_1_. The blood was loaded into a driving syringe. The viscosity of the suspended blood was obtained as *μ*_1_ by supplying the blood into a microfluidic chip. The needle (20 G) was removed from the syringe, and the syringe end was sealed with a plastic cap. The syringe was placed in a centrifuge (Allegra X-30, Beckman Coulter^TM^, Brea, CA, USA) and rotated at 4000 rpm for 10 min. After centrifugation, suspended blood was separated into an RBC-rich layer and RBC-depleted layer (i.e., medium) within the syringe. In the second step, the plastic cap was removed from the syringe, and a needle was attached to the syringe. The blood collected in the plastic cap was then used to determine the hematocrit of the RBC-rich blood (*ϕ*_2_). The syringe was mounted on a syringe pump. By operating the syringe pumps, both types of blood (i.e., RBC-rich blood and RBC-depleted blood) were delivered sequentially into a microfluidic chip. The corresponding viscosities of RBC-rich blood and RBC-depleted blood were then obtained as *μ*_2_ and *μ*_3_, respectively. For simplicity, the hematocrit of the RBC-depleted blood was assumed to be *ϕ*_3_ = 0.

As shown in the middle panel of Figure 1A, a needle was attached to a syringe tip for blood viscosity measurements. The needle was replaced by a plastic cap for separating the suspended blood with a centrifuge. Using a micro-hemocytometer (VS-18000, Vison Science, Daejeon, Republic of Korea), the corresponding hematocrit values of the suspended blood and RBC-rich blood were determined as *ϕ*_1_ and *ϕ*_2_, respectively.

The right-side panel represents the blood viscosity measurement using the coflowing streams method [52,53,54]. To measure blood viscosity in the microfluidic environment, a microfluidic chip consisted of two inlets, a straight channel (width [*w*] = 1000 μm, length (*L*) = 14 mm) and an outlet. As shown in Figure 1A, blood was loaded through the inlet formed at the upper position of the channel. Reference fluid was supplied through the inlet formed at the side position of the channel. Based on microfabrication procedures (i.e., photo-lithography and soft-lithography), a polydimethylsiloxane (PDMS) block was replicated from a silicon master mold. Two inlets were formed using a biopsies punch (outer diameter = 0.75 mm). The PDMS block was bonded on a glass substrate using plasma treatment. To increase the bonding strength substantially, it was exposed to a hot plate of 140 °C for 30 min [55]. After test blood and reference fluid were loaded into each syringe, both syringes were positioned in two syringe pumps. Herein, a glycerin solution (30%) was selected as the reference fluid. As the flow rate of each fluid was set to *Q_b_* (blood) and *Q_r_* (reference fluid), both fluids were delivered into a microfluidic chip. To accurately detect blood viscosity, the interface between the two fluids was relocated near the middle width of the channel (*β* = 0.5) by adjusting the flow rate of the reference fluid. For simple mathematical representation, both streams in a single channel were mathematically represented using a discrete fluidic circuit model, which consisted of the flow rate (*Q_b_*, *Q_r_*) and fluidic resistance (*R_b_*: fluidic resistance of blood; *R_r_*: fluidic resistance of reference fluid). As each stream had the same pressure in a straight channel, the pressure relation between each stream was derived as $P_{r}\approx$ *P_b_*. The *P_r_* and *P_b_* were expressed as *P_r_* = *R_r_* × *Q_r_* and *P_b_* = *R_b_* × *Q_b_*. Using the same pressure condition of each stream (i.e., *R_r_* × *Q_r_* ≈ *R_b_* × *Q_b_*) [52], the viscosity formula of blood (*μ_b_*) was derived as $\mu_{b}=\mu_{r}(\frac{\beta }{1-\beta })(\frac{Q_{r}}{Q_{b}})$. Herein, the *μ_r_* denotes the viscosity of the reference fluid.

As shown in Figure 1B, time-lapse blood viscosity was measured through a two-step procedure. Herein, control blood (*ϕ_vol_* = 0.5) was prepared by adding normal RBCs (500 μL) into 1× PBS (500 μL). The flow rate of the control blood was set to *Q_b_* = 5 mL/h. In the first step, the hematocrit and viscosity of the suspended blood was quantified as *ϕ*_1_ = 0.44 ± 0.01 (*n* = 4) and *μ*_1_ = 2.02 ± 0.11 cP (*n* = 124), respectively. In the second step, the corresponding hematocrit–viscosity of the RBC-rich blood was obtained as *ϕ*_2_ = 0.97 ± 0.01 (*n* = 4) and *μ*_2_ = 17.19 ± 3.25 cP (*n* = 47). The viscosity of RBC-depleted blood was measured as *μ*_3_ = 1.24 ± 0.0 7 cP (*n* = 117).

To find out the regression coefficients of the K-D model, as shown in the left-side panel of Figure 1C, three hematocrit–viscosity datasets are plotted on the hematocrit (*ϕ*) axis and viscosity (*μ*) axis. According to the K-D regression model as $\mu =\mu_{0}{\left(1-\frac{\phi }{\phi_{m}}\right)}^{-\alpha \phi_{m}}$, three unknown coefficients should be identified to get the full hematocrit–viscosity curve. Herein, if the *ϕ_m_* was assumed as *Q_m_* = 1, the remaining two unknown coefficients (i.e., *μ*_0_ and *α*) could be obtained using linear regression analysis. In the K–D model, the *α* could be interpreted as an effective parameter representing the sensitivity of viscosity to hematocrit, analogous to an intrinsic-viscosity-related contribution in suspension theory. For blood samples, the *α* should be regarded as a semi-empirical parameter reflecting the combined effects of RBC properties and intercellular interactions. By taking the natural logarithm of both sides of the K–D model, the K-D regression formula could be rewritten in linearized form as ln (*μ*) = ln (*μ*_0_) − α*ϕ_m_* × ln (1 − *ϕ*/*ϕ_m_*). Horizontal and vertical axes were defined as ln (1 − *ϕ*/*ϕ_m_*) and ln (*μ*). As shown in right-side panel of Figure 1C, three hematocrit–viscosity datasets were then mapped onto the horizontal and vertical axes. According to linear regression analysis, two unknown coefficients (*μ*_0_, α) could be estimated accurately. That is, a full hematocrit–viscosity curve could be obtained using three hematocrit–viscosity datasets.

### 2.2. Blood Sample Preparation

Concentrated erythrocytes were provided by the Gwangju–Chonnam Blood Bank (Gwangju, Republic of Korea) and kept refrigerated until use in the experiment. Based on the established washing method described previously [56], normal RBCs were collected by repeatedly removing the supernatant suspension medium and buffy coat.

First, to determine how hematocrit affects blood viscosity, test suspensions were prepared by mixing normal RBCs with 1× PBS to obtain RBC volume fractions (*ϕ_vol_*) between 10% and 100%. Second, to further examine the effect of the suspending medium, normal RBCs at a constant volume fraction (*ϕ_vol_* = 0.5) were suspended in dextran solutions (*C_dex_* = 5~30 mg/mL). The dextran solutions were made by dissolving dextran powder (*Leuconostoc* spp., MW 450–650 kDa, Sigma-Aldrich, St. Louis, MO, USA) in 1× PBS. Lastly, the effect of RBC rigidity on blood viscosity was investigated by modulating cell stiffness with glutaraldehyde treatment. Two glutaraldehyde concentrations (*C_GA_* = 0.075% and 0.15%) were prepared by diluting a 25% aqueous glutaraldehyde stock solution (Grade II, Sigma-Aldrich, USA) into 1× PBS. Normal RBCs were incubated in each glutaraldehyde solution with stirring for 10 min. Following centrifugation, the fixed RBCs were collected after removal of the supernatant PBS. The final test suspensions were then obtained by resuspending the hardened RBCs into 1× PBS.

### 2.3. Statistical Analysis

Data analysis was conducted using MINITAB software (Version 22.4, Minitab Inc., State College, PA, USA) and Microsoft Excel (Version 365, Microsoft, Redmond, WA, USA). Under the assumption of normality, all measurements were expressed as mean ($\bar{x}$) ± standard deviation (σ). Number of data was denoted as *n*; 95% confidence intervals (CIs) (confidence interval) were determined from $\bar{x}\;-1.96\frac{\;\sigma }{\sqrt{n}}$ to $\bar{x}\;+\;1.96\frac{\;\sigma }{\sqrt{n}}$. Differences among groups were assessed using one-way ANOVA, and statistical significance was accepted at a *p*-value < 0.05. Linear regression analysis was further performed in Microsoft Excel, and the resulting slope and intercept were used to calculate two unknown coefficients of the K-D regression model.

## 3. Results and Discussion

### 3.1. Determination of Maximum Packing Volume Fraction (ϕ_m_) in the K-D Regression Model

In this subsection, the maximum packing volume fraction (*ϕ_m_*) in the K–D regression model was determined by carefully evaluating the regression error using the root-mean-square (RMS) value. Two previously reported datasets were used to identify the best-fitted curve and the corresponding RMS as a function of *ϕ_m_*.

**Figure 2:**
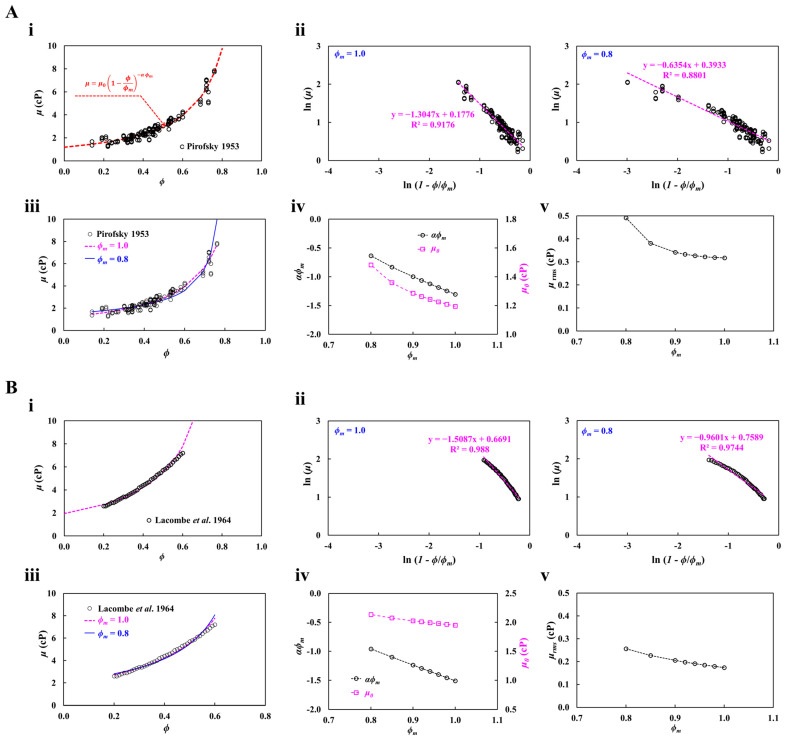
Determination of *ϕ_m_* for the K-D regression model using the hematocrit–viscosity set taken from two studies. (**A**) The impact of *ϕ_m_* on the accuracy of the regression model using hematocrit–viscosity sets taken from the literature (Pirofsky [57]). (**i**) Variations in blood viscosity (*μ*) with respect to hematocrit (*ϕ*). The red curve represents the best-fitted curve of the K-D regression model. (**ii**) Linear curve-fitting procedure with respect to *ϕ_m_* = 1.0 and 0.8. (**iii**) Comparison of a curve-fitting formula with respect to *ϕ_m_*. The blue and pink lines denote the best-fitted curve obtained by assuming *ϕ_m_* = 0.8 and 1.0. (**iv**) Variations in *αϕ_m_* and *μ*_0_ with respect to *ϕ_m_*. (**v**) Variations in root-mean-square value of *μ* (*μ_rms_*) with respect to *ϕ_m_*. (**B**) The effect of *ϕ_m_* on K-D model accuracy using hematocrit–viscosity data taken from the literature (Lacombe et al. [58]). (**i**) Variations in *μ* with respect to *ϕ*. (**ii**) Linear curve-fitting procedure with respect to *ϕ_m_* = 1.0 and 0.8. According to the linear regression analysis, the corresponding formula of each *ϕ_m_* was obtained as ln (*μ*) = −1.5087 ln (1 − *ϕ*/*ϕ_m_*) + 0.6691 (R^2^ = 0.988) for *ϕ_m_* = 1.0 and ln (*μ*) = −0.9601 ln (1 − *ϕ*/*ϕ_m_*) + 0.7589 (R^2^ = 0.8801) for *ϕ_m_* = 0.8. (**iii**) Comparison of the best-fitted curve with respect to *ϕ_m_* = 0.8 and 1.0. (**iv**) Variations in *αϕ_m_* and *μ*_0_ with respect to *ϕ_m_*. (**v**) Variations in *μ_rms_* with respect to *ϕ_m_*.

First, using hematocrit–viscosity data taken from reference (Pirofsky) [57], as shown in Figure 2(Ai), viscosity (*μ*) and (*ϕ*) are plotted along the horizontal and vertical axes, respectively. The red line represents the best-fitted curve determined by linear regression analysis. Figure 2(Aii) shows the best-fitted curve with respect to *ϕ_m_* = 1.0 and 0.8. Herein, the horizontal and vertical axes were defined as ln (1 − *ϕ*/*ϕ_m_*) and ln (*μ*), respectively. Hematocrit–viscosity datasets are plotted along both axes. According to the linear regression analysis, the corresponding formular of each *ϕ_m_* was obtained as ln (*μ*) = −1.3047 ln (1 − *ϕ*/*ϕ_m_*) + 0.1776 (R^2^ = 0.9176) for *ϕ_m_* = 1.0 and ln (*μ*) = −0.6384 ln (1 − *ϕ*/*ϕ_m_*) + 0.3933 (R^2^ = 0.8801) for *ϕ_m_* = 0.8. That is, the corresponding *αϕ_m_* of each *ϕ_m_* was obtained as *αϕ_m_* = −1.3047 (*ϕ_m_* = 1.0) and *αϕ_m_* = −0.6387 (*ϕ_m_* = 0.8). In addition, the corresponding *μ*_0_ of each *ϕ_m_* was obtained as *μ*_0_ = 1.194 cP (*ϕ_m_* = 1.0) and *μ*_0_ = 1.482 cP (*ϕ_m_* = 1.0). As the coefficient of R^2^ was obtained as high value of R^2^ = 0.8801~0.9176, both best-fitted curves represented variations in *μ* with respect to *ϕ* sufficiently. As shown in Figure 2(Aiii), the raw data and best-fitted curves are superimposed along the hematocrit (*ϕ*) and viscosity (*μ*) axes, respectively, where blue and pink lines represent the best-fitted curves obtained by assuming *ϕ_m_* = 0.8 and *ϕ_m_* = 1.0, respectively. The results suggest that the best-fitted curve obtained by assuming *ϕ_m_* = 1.0 was more accurate than that obtained by assuming *ϕ_m_* = 0.8. To assess the effect of *ϕ_m_* on the accuracy of the regression analysis, multiple data points of *ϕ_m_* were selected between 0.8 and 1.0.

As shown Figure 2(Aiv), linear regression analysis yielded the changes in *αϕ_m_* and *μ*_0_ as a function of *ϕ_m_*. According to the results, as *ϕ_m_* increased from 0.8 to 1.0, *αϕ_m_* and *μ*_0_ tended to decrease gradually, confirming that the *ϕ_m_* had a strong impact on determination on both coefficients. In addition, to evaluate the accuracy of the regression analysis, the root-mean-square (RMS) value of *μ* (i.e., *μ_rms_*) was calculated with respect to *ϕ_m_*. Herein, the *μ_rms_* represented the average deviation between measured and predicted values (i.e., $\mu_{rms}=\sqrt{\frac{1}{n}\sum_{i=1}^{i=n}{\left(\mu_{i}-{\hat{\mu }}_{i}\right)}^{2}}$; *μ_i_*: measured viscosity and ${\hat{\mu }}_{i}$: predicted viscosity). As shown in Figure 2(Av), the variations in *μ_rms_* are plotted as a function of *ϕ_m_*. The results indicated that a higher value of *ϕ_m_* led to a substantial decrease in *μ_rms_*. Accordingly, the most accurate curve fitting was obtained when *ϕ_m_* was set to 1.0.

Second, hematocrit–viscosity data taken from the literature (Lacombe et al.) [58] was additionally used to validate the contribution of *ϕ_m_* to the accuracy of the regression analysis. Figure 2(Bi) depicts all hematocrit–viscosity datasets and the best-fitted curve of the K-D regression model. As shown in Figure 2(Bii), according to the linear regression analysis, the best-fitted curve was obtained by assuming *ϕ_m_* = 1.0 and *ϕ_m_* = 0.8. As a result, the corresponding formula of each *ϕ_m_* was obtained as ln (*μ*) = −1.5087 ln (1 − *ϕ*/*ϕ_m_*) + 0.6691 (R^2^ = 0.988) for *ϕ_m_* = 1.0, and ln (*μ*) = −0.9601 ln (1 − *ϕ*/*ϕ_m_*) + 0.7589 (R^2^ = 0.8801) for *ϕ_m_* = 0.8. That is, the corresponding *αϕ_m_* of each *ϕ_m_* was estimated as *αϕ_m_* = −1.5087 (*ϕ_m_* = 1.0) and *αϕ_m_* = −0.9601 (*ϕ_m_* = 0.8). Additionally, the corresponding *μ*_0_ of each *ϕ_m_* was estimated as *μ*_0_ = 1.952 cP (*ϕ_m_* = 1.0) and *μ*_0_ = 2.136 cP (*ϕ_m_* = 0.8). To validate the accuracy of the best-fitted curve, as shown in Figure 2(Biii), experimental datasets and both regression curves were superimposed on the hematocrit (*ϕ*) and viscosity (*μ*) axes. The results indicated that both best-fitted curves were well matched with the experimental datasets. To investigate the contribution of *ϕ_m_* to the coefficients (i.e., *αϕ_m_* and *μ*_0_), multiple values of *ϕ_m_* were selected between 0.8 and 1.0. As shown in Figure 2(Biv), variations in *αϕ_m_* and *μ*_0_ were obtained with respect to *ϕ_m_*. The results indicated that the *ϕ_m_* contributed to decreasing both coefficients substantially. Specifically, the *ϕ_m_* had a strong influence on the determination of *αϕ_m_* and *μ*_0_. As shown in Figure 2(Bv), to validate the accuracy of the best-fitted curve, variations in *μ_rms_* were obtained with respect to *ϕ_m_*. According to the results, the *μ_rms_* tended to decrease gradually at a higher value of *ϕ_m_*. Thus, it is necessary to set *ϕ_m_* = 1.0 for getting a more accurate best-fitted curve.

From the curve-fitting analysis of two kinds of previous hematocrit–viscosity datasets, the maximum packing volume fraction (*ϕ_m_*) in the K–D regression model had a strong influence on the determination of *αϕ_m_* and *μ*_0_. Additionally, the root-mean-square value of the best-fitted curve was minimized at a higher value of *ϕ_m_* = 1.0. Based on the simulation study, the study adopted *ϕ_m_* = 1.0 for getting a best-fitted curve of the hematocrit–viscosity datasets.

From the curve-fitting analysis of two previously reported hematocrit–viscosity datasets, the maximum packing volume fraction (*ϕ_m_*) in the K–D regression model was found to strongly affect the determination of *αϕ_m_* and *μ*_0_. In addition, the root-mean-square error of the fitted curve was minimized when a higher value of *ϕ_m_* = 1.0 was used. Based on this simulation analysis, *ϕ_m_* = 1.0 was adopted to obtain the best-fitted hematocrit–viscosity curve.

### 3.2. Hematocrit–Viscosity Measurement Datasets of Control Blood

In this subsection, with regard to control blood, full hematocrit–viscosity datasets were obtained at higher shear rates (i.e., $\dot{\gamma }$ > $10^{3}$ s^−1^). Herein, the control blood was prepared by adding normal RBCs into 1× PBS.

**Figure 3:**
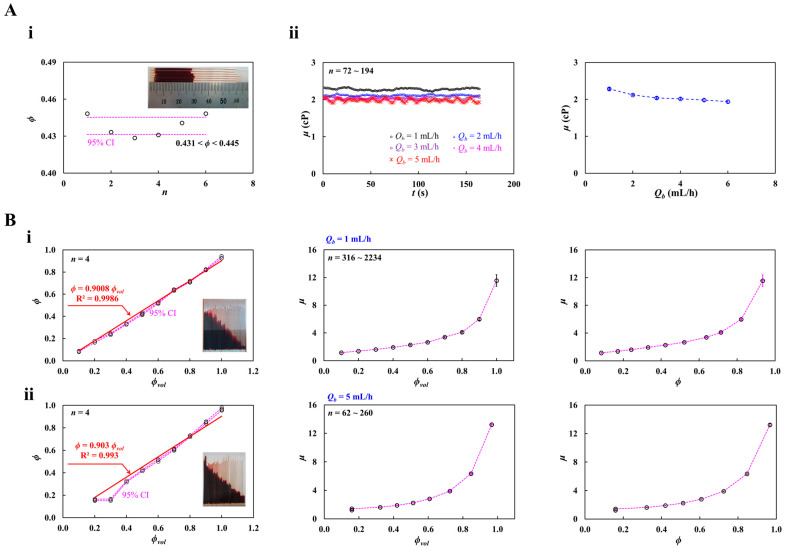
Experimental measurement of the hematocrit–viscosity datasets of control blood at a high shear rate. The control blood was prepared by adding normal RBCs into 1× PBS. (**A**) Determination of blood flow rate (*Q_b_*). Herein, the RBC volume fraction in the blood was set to *ϕ_vol_* = 0.5. (**i**) Variations in hematocrit (*ϕ*) for six control blood samples (*S_n_* = 6). The dashed line denotes 95% confidential intervals (i.e., 0.431 < *ϕ* < 0.445). (**ii**) Variations in μ with respect to the blood flow rate (*Q_b_*). The left-side panel shows time-lapse viscosity with respect to *Q_b_* ranging from Q*_b_* = 1 mL/h to *Q_b_* = 6 mL/h. The right-side panel shows variations in *μ* with respect to *Q_b_*. The error bar represents the standard deviation. (**B**) Measurement of hematocrit–viscosity datasets at *Q_b_* = 1 and 5 mL/h. (**i**) Measurement of *ϕ*-*μ* obtained at *Q_b_* = 1 mL/h. The left-side panel shows the relationship between *ϕ* and *ϕ_vol_*. The dashed line exhibits the 95% CI. The mid-side panel exhibits variations in *μ* with respect to *ϕ_vol_*. The error bar denotes the standard deviation. The right-side panel shows variations in *μ* with respect to *ϕ*. The error bar represents the standard deviation. (**ii**) Measurement of *ϕ*-*μ* obtained at *Q_b_* = 5 mL/h. The left-side panel shows the relationship between *ϕ* and *ϕ_vol_*. The dashed line exhibits the 95% CI. The mid-side panel exhibits variations in *μ* with respect to *ϕ_vol_*. The error bar denotes the standard deviation. The right-side panel shows variations in *μ* with respect to *ϕ*. The error bar represents standard deviation.

First, to determine the appropriate blood flow rate of the syringe pump (*Q_b_*), blood viscosity (*μ*) was measured over a flow rate ranging from *Q_b_* = 1 mL/h to *Q_b_* = 5 mL/h. Following the previously reported coflowing streams method [52], the reference-fluid flow rate (*Q_r_*) was adjusted so that the interface was positioned near the center of the channel width. The RBC volume fraction in the blood was set to *ϕ_vol_* = 0.5. As shown in Figure 3(Ai), six control blood samples (*S_n_* = 6) were set to probe the variation in hematocrit (*ϕ*). The inset exhibits capillary tubes used for measuring hematocrit. Dashed lines represent the 95% CI (i.e., 0.431 < *ϕ* < 0.445). Next, the blood viscosity of control blood was obtained with respect to *Q_b_*. To relocate the interface near the center of the channel width, with respect to *Q_b_*, the corresponding flow rate of the reference fluid was set to *Q_r_* = 1 mL/h (*Q_b_* = 1 mL/h), *Q_r_* = 2 mL/h (*Q_b_* = 2 mL/h), *Q_r_* = 2.5 mL/h (*Q_b_* = 3 mL/h), *Q_r_* = 4 mL/h (*Q_b_* = 4 mL/h), *Q_r_* = 4.2 mL/h (*Q_b_* = 5 mL/h), and *Q_r_* = 5 mL/h (*Q_b_* = 6 mL/h). The corresponding interface of both flow rate conditions was measured as *β* = 0.460 ± 0.004 (*Q_b_* = 1 mL/h, *Q_r_* = 1 mL/h), *β* = 0.442 ± 0.002 (*Q_b_* = 2 mL/h, *Q_r_* = 2 mL/h), *β* = 0.477 ± 0.003 (*Q_b_* = 3 mL/h, *Q_r_* = 2.5 mL/h), *β* = 0.462 ± 0.003 (*Q_b_* = 4 mL/h, *Q_r_* = 4 mL/h), *β* = 0.468 ± 0.005 (*Q_b_* = 5 mL/h, *Q_r_* = 4.2 mL/h), and *β* = 0.464 ± 0.005 (*Q_b_* = 6 mL/h, *Q_r_* = 5 mL/h). As shown in the left-side panel of Figure (3Aii), the blood viscosity of control blood was obtained with respect to *Q_b_*. The corresponding viscosity of each flow rate was obtained as *μ* = 2.29 ± 0.03 cP (*n* = 164, *Q_b_* = 1 mL/h), *μ* = 2.12 ± 0.02 cP (*n* = 189, *Q_b_* = 2 mL/h), *μ* = 2.04 ± 0.03 cP (*n* = 194, *Q_b_* = 3 mL/h), *μ* = 2.02 ± 0.03 cP (*n* = 108, *Q_b_* = 4 mL/h), *μ* = 1.98 ± 0.04 cP (*n* = 181, *Q_b_* = 5 mL/h), and *μ* = 1.94 ± 0.04 cP (*n* = 74, *Q_b_* = 6 mL/h). Based on the shear rate formula [52] (i.e., $\dot{\gamma }=\frac{6\;Q_{b}}{\beta \;w\;h^{2}})$, the corresponding shear rate of each blood flow rate was estimated as $\dot{\gamma }\;$=1451.5 s^−1^ (*Q_b_* = 1 mL/h), $\dot{\gamma }\;$= 3020 s^−1^ (*Q_b_* = 2 mL/h), $\dot{\gamma }\;$= 4194.1 s^−1^ (*Q_b_* = 3 mL/h), $\dot{\gamma }\;$= 5773.2 s^−1^ (*Q_b_* = 4 mL/h), $\dot{\gamma }\;$= 7130.1 s^−1^ (*Q_b_* = 5 mL/h), and $\dot{\gamma }\;$= 8617.7 s^−1^ (*Q_b_* = 6 mL/h). The right-side panel of Figure 3(Aii) exhibits variations in blood viscosity with respect to *Q_b_*. Blood viscosity decreased substantially over *Q_b_* = 1~3 mL/h, whereas only a slight decrease was observed over *Q_b_* = 3~6 mL/h. Based on these results, *Q_b_* = 1 and 5 mL/h were selected for measuring blood viscosity at higher shear rates.

Second, hematocrit–viscosity datasets were obtained at *Q_b_* = 1 and 5 mL/h. As shown in Figure 3(Bi), the hematocrit–viscosity datasets were obtained at *Q_b_* = 1 mL/h. The left-side panel shows the relationship between *ϕ* and *ϕ_vol_*. Herein, the *ϕ_vol_* of control blood was adjusted to *ϕ_vol_* = 0.1~1.0. The corresponding hematocrit of each type of blood was obtained with a micro-hemocytometer. The dashed line exhibits the 95% CI. The inset shows representative images used to determine the RBC volume fraction (*ϕ*) in a microcapillary tube following centrifugation. According to the linear regression analysis, the best-fitted curve was obtained as *ϕ* = 0.908 *ϕ_vol_* (R^2^ = 0.9986). The middle-side panel exhibits variations in *μ* with respect to *ϕ_vol_*. The number of viscosity data at each *ϕ_vol_* ranged from *n* = 316 to *n* = 2234. The right-side panel shows variations in *μ* with respect to *ϕ*. The corresponding viscosity of each hematocrit was obtained as *μ* = 1.14 ± 0.03 cP (*ϕ* = 0.08 ± 0.002), *μ* = 1.37 ± 0.03 cP (*ϕ* = 0.17 ± 0.006), *μ* = 1.60 ± 0.04 cP (*ϕ* = 0.24 ± 0.006), *μ* = 1.92 ± 0.05 cP (*ϕ* = 0.33 ± 0.004), *μ* = 2.26 ± 0.05 cP (*ϕ* = 0.42 ± 0.007), *μ* = 2.66 ± 0.08 cP (*ϕ* = 0.52 ± 0.005), *μ* = 3.38 ± 0.08 cP (*ϕ* = 0.64 ± 0.005), *μ* = 4.10 ± 0.12 cP (*ϕ* = 0.71 ± 0.006), *μ* = 5.99 ± 0.18 cP (*ϕ* = 0.82 ± 0.003), and *μ* = 11.56 ± 0.83 cP (*ϕ* = 0.93 ± 0.011). On the other hand, as shown in Figure 3(Bii), hematocrit–viscosity datasets were obtained at *Q_b_* = 5 mL/h. The left-side panel shows the relationship between *ϕ* and *ϕ_vol_*. According to the linear regression analysis, the best-fitted curve was obtained as *ϕ* = 0.903 *ϕ_vol_* (R^2^ = 0.993). The middle-side panel exhibits variations in *μ* with respect to *ϕ_vol_*. The number of viscosity data at each *ϕ_vol_* ranged from *n* = 52 to *n* = 260. The right-side panel shows variations in *μ* with respect to *ϕ*. The corresponding viscosity of each hematocrit was obtained as *μ* = 1.24 ± 0.02 cP (*ϕ* = 0.16 ± 0.006), *μ* = 1.43 ± 0.02 cP (*ϕ* = 0.16 ± 0.007), *μ* = 1.63 ± 0.02 cP (*ϕ* = 0.32 ± 0.004), *μ* = 1.89 ± 0.03 cP (*ϕ* = 0.42 ± 0.006), *μ* = 2.24 ± 0.03 cP (*ϕ* = 0.51 ± 0.008), *μ* = 2.80 ± 0.04 cP (*ϕ* = 0.61 ± 0.006), *μ* = 3.89 ± 0.08 cP (*ϕ* = 0.72 ± 0.007), *μ* = 6.34 ± 0.16 cP (*ϕ* = 0.85 ± 0.009), and *μ* = 13.23 ± 0.26 cP (*ϕ* = 0.97 ± 0.011).

Experimental measurements provided two hematocrit–viscosity datasets at *Q_b_* = 1 and 5 mL/h, which were used to validate the accuracy of the proposed method for reconstructing the full hematocrit–viscosity curve based on the K–D regression model.

### 3.3. Quantitative Evaluation of Proposed Method Using Hematocrit–Viscosity Datasets of Control Blood

In this subsection, as shown in Figure 3B, the proposed method for getting the full hematocrit–viscosity curve was validated using experimental data. Herein, with respect to each hematocrit, blood viscosity was obtained at *Q_b_* = 1 and 5 mL/h.

**Figure 4:**
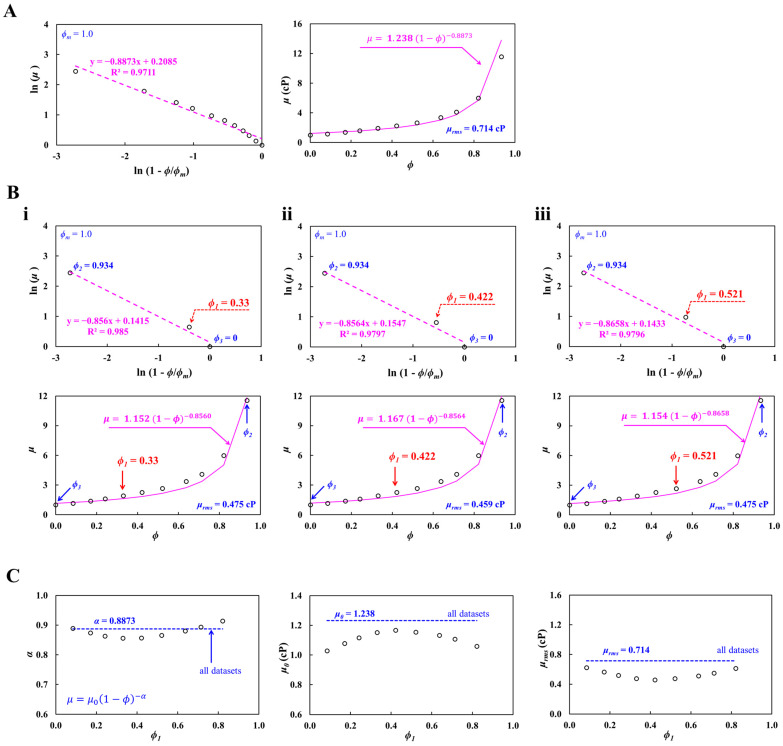
Validation of the proposed method using eleven hematocrit–viscosity sets, where blood viscosity was measured at a flow rate of *Q_b_* = 1 mL/h. (**A**) Complete hematocrit–viscosity curve identification using eleven *ϕ*-*μ* datasets. As shown in the left-side panel, hematocrit–viscosity datasets are mapped on the ln (1 − *ϕ*/*ϕ_m_*) and ln (*μ*) axes. The right-side panel shows all hematocrit–viscosity datasets and their best-fitted curve of $\mu =1.238\;{\left(1-\phi \right)}^{-0.8873}$. (**B**) Regression formula using three *ϕ*-*μ* datasets. Herein, both boundary datasets (i.e., *ϕ*_2_ = 0.934: *μ*_2_ = 11.56 cP and *ϕ*_3_ = 0: *μ*_3_ = 1 cP) are fixed to get the complete hematocrit–viscosity curve. The middle point dataset (*ϕ*_1_) was selected from the remaining nine datasets. (**i**) Full hematocrit–viscosity curve using the midpoint dataset (i.e., *ϕ*_1_ = 0.33, *μ*_1_ = 1.917 cP). The complete hematocrit–viscosity curve and root-mean-square error were obtained as $\mu =1.152\;{\left(1-\phi \right)}^{-0.8560}$ and *μ_rms_* = 0.475 cP. (**ii**) Full hematocrit–viscosity curve using midpoint dataset (i.e., *ϕ*_1_ = 0.422, *μ*_1_ = 2.264 cP). Complete hematocrit–viscosity curve and root-mean-square error were estimated as $\mu =1.167\;{\left(1-\phi \right)}^{-0.8564}$ and *μ_rms_* = 0.459 cP. (**iii**) Full hematocrit–viscosity curve using midpoint dataset (i.e., *ϕ*_1_ = 0.521, *μ*_1_ = 2.657 cP). Full hematocrit–viscosity curve and root-mean-square error were calculated as $\mu =1.154\;{\left(1-\phi \right)}^{-0.8658}$ and *μ_rms_* = 0.475 cP. (**C**) Quantitative comparison of full hematocrit–viscosity curve obtained from all datasets (i.e., conventional method) and three selected datasets (i.e., proposed method). The dashed line represents *α* = 0.8873, *μ*_0_ = 1.238 cP, and *μ_rms_* = 0.714 cP obtained from all hematocrit–viscosity datasets. The left-side panel exhibits variations in α with respect to the selected midpoint dataset (*ϕ*_1_). The mid-side panel shows variations in *μ*_0_ with respect to the selected midpoint dataset (*ϕ*_1_). The right-side panels depict variations in *μ_rms_* with respect to the midpoint dataset (*ϕ*_1_).

First, validation of proposed method was conducted using eleven hematocrit–viscosity datasets, where blood viscosity was measured at a flow rate of *Q_b_* = 1 mL/h ($\dot{\gamma }\;$= 1451.5 s^−1^). Figure 4A represents the complete hematocrit–viscosity curve identification using eleven *ϕ*-*μ* datasets. Herein, the *ϕ_m_* was set to *ϕ_m_* = 1.0. As shown in the left-side panel, hematocrit–viscosity datasets are mapped on the ln (1 − *ϕ*/*ϕ_m_*) and ln (*μ*) axes. According to the linear regression analysis, the best-fitted curve was obtained as ln (*μ*) = −0.8873 ln (1 − *ϕ*/*ϕ_m_*) + 0.2085 (R^2^ = 0.9711). From the curve-fitting formula, *αϕ_m_* and *μ*_0_ were obtained as *αϕ_m_* = −0.8873 and *μ*_0_ = 1.238 cP, respectively. As shown in right-side panel, the best-fitted curve of $\mu =1.238\;{\left(1-\phi \right)}^{-0.8873}$ is superimposed on all hematocrit–viscosity datasets. The results showed that the best-fitted curve was in good agreement with the experimental datasets, except at the highest hematocrit value of *ϕ* = 0.934. The root-mean-square error was estimated as *μ_rms_* = 0.714 cP. Using all experimental datasets as shown in Figure 4A, the full hematocrit–viscosity curve was identified using three *ϕ*-*μ* datasets. Herein, both boundary datasets (i.e., *ϕ*_2_ = 0.934: *μ*_2_ = 11.56 cP and *ϕ*_3_ = 0: *μ*_3_ = 1 cP) were fixed during hematocrit–viscosity curve identification. The middle point dataset (*ϕ*_1_ = 0.08~0.82) was only selected from the remaining nine datasets. As show in Figure 4(Bi), the full hematocrit–viscosity curve was calculated using midpoint dataset (i.e., *ϕ*_1_ = 0.33, *μ*_1_ = 1.917 cP). According to the linear regression analysis, the best-fitted curve was obtained as ln (*μ*) = −0.856 ln (1 − *ϕ*) + 0.1415 (R^2^ = 0.985); *α* and *μ*_0_ were estimated as *α* = −0.856 and *μ*_0_ = 1.152 cP, respectively. The complete hematocrit–viscosity curve was then identified as $\mu =1.152\;{\left(1-\phi \right)}^{-0.856}$. Additionally, root-mean-square error was estimated as *μ_rms_* = 0.475 cP. Similarly, as shown in Figure 4(Bii), the full hematocrit–viscosity curve was estimated using the midpoint dataset (i.e., *ϕ*_1_ = 0.422, *μ*_1_ = 2.264 cP). According to the linear regression analysis, the best-fitted curve was obtained as ln (*μ*) = −0.8564 ln (1 − *ϕ*) + 0.1547 (R^2^ = 0.9797); *α* and *μ*_0_ were estimated as *α* = −0.8564 and *μ*_0_ = 1.167 cP, respectively. The complete hematocrit–viscosity curve was then identified as $\mu =1.167\;{\left(1-\phi \right)}^{-0.8564}$. The root-mean-square error was estimated as *μ_rms_* = 0.459 cP. As shown in Figure 4(Biii), the full hematocrit–viscosity curve was calculated using the midpoint dataset (i.e., *ϕ*_1_ = 0.521, *μ*_1_ = 2.657 cP). According to the linear regression analysis, the best-fitted curve was obtained as ln (*μ*) = −0.8658 ln (1 − *ϕ*) + 0.1433 (R^2^ = 0.9796); *α* and *μ*_0_ were estimated as *α* = −8658 and *μ*_0_ = 1.154 cP, respectively. The full hematocrit–viscosity curve was then identified as $\mu =1.154\;{\left(1-\phi \right)}^{-0.8658}$. The root-mean-square error was estimated as *μ_rms_* = 0.475 cP. Figure 4C exhibits a quantitative comparison of the full hematocrit–viscosity curve obtained by all datasets and three datasets selected from all datasets. The left-side panel exhibits variations in *α* with respect to the selected midpoint dataset (*ϕ*_1_). The dashed line represents α = 0.8873, obtained by all hematocrit–viscosity datasets. The value of α was strongly affected by the selected midpoint dataset. The mid-side panel shows variations in *μ*_0_ with respect to the selected midpoint dataset (*ϕ*_1_). The dashed line represents *μ*_0_ = 1.238 cP, obtained by all hematocrit–viscosity datasets. Considering that the blood viscosity at *ϕ* = 0 was measured as *μ* = 1.028 cP, the *μ*_0_ estimated from all hematocrit–viscosity datasets was overestimated by approximately 20.4%. Moreover, the *μ*_0_ values obtained from the three selected datasets were lower than those derived from the full dataset, with minimum values at low and high hematocrit and a maximum value near ϕ = 0.33–0.52. The right-side panels depict variations in *μ_rms_* with respect to the midpoint dataset (*ϕ*_1_). The dashed line represents *μ_rms_* = 0.714 cP obtained by all hematocrit–viscosity datasets. The proposed method using three selected datasets yielded a lower root-mean-square error than the conventional method using all datasets. In particular, when *ϕ* was selected between 0.33 and 0.52, the root-mean-square error was estimated as *μ_rms_* = 0.475~0.495 cP. These results indicated that the full hematocrit–viscosity curve obtained from the three selected datasets was more accurate and remained in good agreement with the overall hematocrit–viscosity datasets.

**Figure 5:**
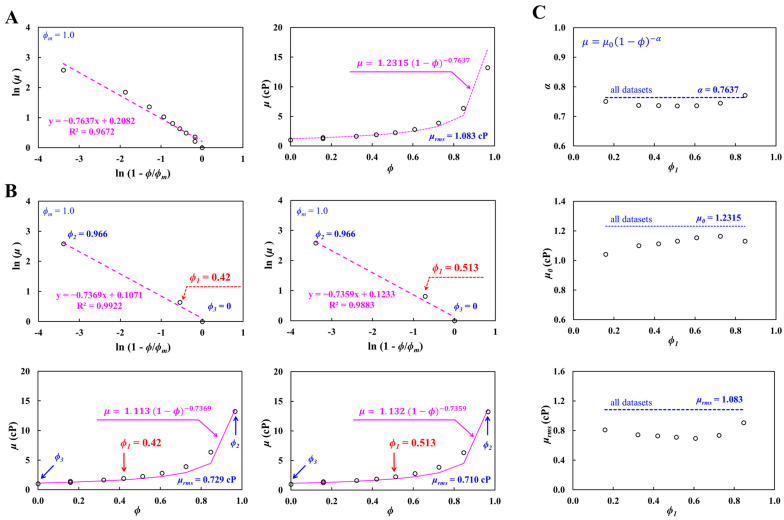
Validation of proposed method using ten hematocrit–viscosity datasets, where blood viscosity was measured at *Q_b_* = 5 mL/h. (**A**) Full hematocrit–viscosity curve identification using ten *ϕ*-*μ* datasets. As shown in the left-side panel, linear regression analysis gave the best-fitted curved as ln (*μ*) = −0.7637 ln (1 − *ϕ*) + 0.2082 (R^2^ = 0.9672). As depicted in the right-side panel, the full hematocrit–viscosity curve of $\mu =1.2315{\left(1-\phi \right)}^{-0.7637}$ was superimposed on all hematocrit–viscosity datasets. Root-mean-square error was estimated as *μ_rms_* = 1.083 cP. (**B**) Full hematocrit–viscosity curve identification using three *ϕ*-*μ* datasets. Herein, both boundary datasets (i.e., *ϕ*_2_ = 0.966: *μ*_2_ = 13.23 cP and *ϕ*_3_ = 0: *μ*_3_ = 1 cP) were fixed during the identification of the complete hematocrit–viscosity curve. First, with regard to the midpoint dataset (i.e., *ϕ*_1_ = 0.42, *μ*_1_ = 1.889 cP), the full hematocrit–viscosity curve and root-mean-square error were obtained as $\mu =1.113\;{\left(1-\phi \right)}^{-0.7369}$ and *μ_rms_* = 0.729 cP. Second, with regard to the midpoint dataset (i.e., *ϕ*_1_ = 0.513, *μ*_1_ = 2.245 cP), the full hematocrit–viscosity curve and root-mean-square error were obtained as $\mu =1.132\;{\left(1-\phi \right)}^{-0.7359}$ and *μ_rms_* = 0.71 cP. (**C**) Quantitative comparison of full hematocrit–viscosity curve obtained by all datasets and three datasets. The dashed line represents *α* = 0.7637, *μ*_0_ = 1.2315 cP, and *μ_rms_* = 1.083 cP obtained from all hematocrit–viscosity datasets. Upper-side panel exhibits variations in *α* with respect to the midpoint dataset (*ϕ*_1_). The mid-side panel shows variations in *μ*_0_ with respect to the midpoint dataset (*ϕ*_1_). The lower-side panels depict variations in *μ_rms_* with respect to the midpoint dataset (*ϕ*_1_).

Second, the full hematocrit–viscosity curve was estimated using ten hematocrit–viscosity datasets, where blood viscosity was measured at *Q_b_* = 5 mL/h ($\dot{\gamma }\;$= 7130.1 s^−1^). Figure 5A depicts the full hematocrit–viscosity curve obtained from ten *ϕ*-*μ* datasets. Herein, *ϕ_m_* was set to *ϕ_m_* = 1.0. As shown in the left-side panel, the linear regression analysis gave best-fitted curve as ln (*μ*) = −0.7637 ln (1 − *ϕ*) + 0.2082 (R^2^ = 0.9672); *α* and *μ*_0_ were then obtained as *α* = 0.7637 and *μ*_0_ = 1.2318 cP. As shown in the right-side panel, the full hematocrit–viscosity curve of $\mu =1.2315\;{\left(1-\phi \right)}^{-0.7637}$ was superimposed on all hematocrit–viscosity datasets. The results showed that the best-fitted curve was in satisfactory agreement with the experimental datasets. The root-mean-square error was estimated as *μ_rms_* = 1.083 cP.

As shown in Figure 5B, the full hematocrit–viscosity curve identification was obtained from the three selected *ϕ*-*μ* datasets. In the identification of the complete hematocrit–viscosity curve, both boundary datasets (i.e., *ϕ*_2_ = 0.966: *μ*_2_ = 13.23 cP and *ϕ*_3_ = 0: *μ*_3_ = 1 cP) were fixed. The midpoint dataset (*ϕ*_1_) was selected from the remaining eight datasets. As shown in left-side panel, with regard to the midpoint dataset (i.e., *ϕ*_1_ = 0.42, *μ*_1_ = 1.889 cP), the best-fitted curve was obtained as ln (*μ*) = −0.7369 ln (1 − *ϕ*) + 0.1071 (R^2^ = 0.9922); *α* and *μ*_0_ were then obtained as *α* = 0.7369 and *μ*_0_ = 1.113 cP. The full hematocrit–viscosity curve and root-mean-square error were obtained as $\mu =1.113{\left(1-\phi \right)}^{-0.7369}$ and *μ_rms_* = 0.729 cP. As shown in the right-side panel, with regard to the midpoint dataset (i.e., *ϕ*_1_ = 0.513, *μ*_1_ = 2.245 cP), the best-fitted curve was obtained as ln (*μ*) = −0.7359 ln (1 − *ϕ*) + 0.1233 (R^2^ = 0.9883). The full hematocrit–viscosity curve and root-mean-square error were obtained as $\mu =1.132{\left(1-\phi \right)}^{-0.7359}$ and *μ_rms_* = 0.71 cP. As shown in Figure 5C, the coefficients of the K-D regression model (*α*, *μ*_0_) and root-mean-square error (*μ_rms_*) are plotted as a function of the selected midpoint dataset (*ϕ*_1_). The dashed line represents *α* = 0.7637, *μ*_0_ = 1.2315 cP, and *μ_rms_* = 1.083 cP obtained from all hematocrit–viscosity datasets. The upper-side panel exhibits variations in *α* with respect to the midpoint dataset (*ϕ*_1_). The results revealed a strong dependence of *α* on *ϕ*_1_. The mid-side panel shows variations in *μ*_0_ with respect to the midpoint dataset (*ϕ*_1_). The *μ*_0_ obtained from the three selected datasets was lower than that derived from the full dataset. The lower-side panels depict variations in *μ_rms_* with respect to the midpoint dataset (*ϕ*_1_). According to the results, the *μ_rms_* obtained from the three selected datasets was much lower than that derived from the full dataset.

From the identification of the full hematocrit–viscosity curve, the exponent of the K-D model was found to depend strongly on the midpoint dataset (*ϕ*_1_, *μ*_1_). The *μ*_0_ and *μ_rms_* values obtained from the three selected datasets were substantially lower than those obtained from all the hematocrit–viscosity datasets. Although the high-hematocrit condition of the RBC-rich blood was beyond the normal physiological range, it was intentionally included as an upper-bound condition generated by centrifugation to provide a boundary dataset for K–D model fitting and to improve reconstruction of the full hematocrit–viscosity relationship. Therefore, the full hematocrit–viscosity curve reconstructed from the three selected datasets could be considered more accurate when compared with the full hematocrit–viscosity curve obtained from all the hematocrit–viscosity curves.

### 3.4. Contribution of Suspending Medium to Full Hematocrit–Viscosity Curve

In this subsection, to evaluate the contribution of the suspending medium to the full hematocrit–viscosity curve [45,59], a specific concentration of a dextran solution (*C_dex_* = 0~30 mg/mL) was prepared as the blood medium. Herein, *C_dex_* = 0 denoted 1× PBS. Test blood (*ϕ_vol_* = 0.5) was then prepared by adding normal RBCs into a specific concentration of the dextran solution. Blood viscosity was obtained at a flow rate of 5 mL/h.

**Figure 6:**
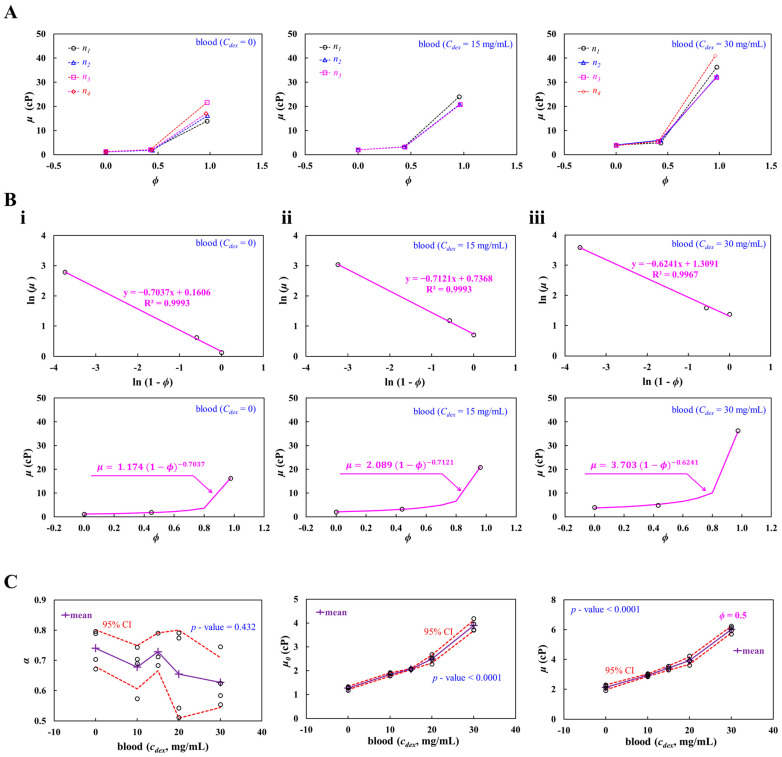
Contribution of blood medium to the full hematocrit–viscosity curve. Herein, test blood was prepared by adding normal RBCs into a specific concentration of the dextran solution (*C_dex_* = 5~30 mg/mL). Blood viscosity was obtained at a flow rate of 5 mL/h. (**A**) Three hematocrit–viscosity datasets with respect to *C_dex_* = 0, 15, and 30 mg/mL. (**B**) Full hematocrit–viscosity curve identification with respect to *C_dex_*. (**i**) Linear regression and full hematocrit–viscosity curve for blood (*C_dex_* = 0). (**ii**) Linear regression and full hematocrit–viscosity curve for blood (*C_dex_* = 15 mg/mL). (**iii**) Linear regression and full hematocrit–viscosity curve for blood (*C_dex_* = 30 mg/mL). (**C**) Contribution of dextran solution to *α*, *μ*_0_, and *μ* (*ϕ* = 0.5). The left-side panel shows variations in α with respect to *C_dex_*. The red-dashed line represents the 95% CI. According to statistical analysis (ANOVA-test), the *p*-value was obtained as 0.432. The mid-side panel depicts variations in *μ*_0_ with respect to *C_dex_*. The ANOVA test gave a *p*-value < 0.0001. The right-side panel shows variations in *μ* (*ϕ* = 0.5) with respect to *C_dex_*. The ANOVA test provided a *p*-value < 0.0001.

As shown in Figure 6A, three hematocrit–viscosity datasets are presented for *C_dex_* = 0, 15, and 30 mg/mL. The left, middle, and right panels show the datasets for *C_dex_* = 0, 15, and 30 mg/mL, respectively. The number of test blood samples was set to *S_n_* = 3~4.

Using the hematocrit–viscosity datasets with respect to the test blood (*C_dex_*), linear regression analysis was conducted to find out the coefficients of the K-D regression model (i.e., α, and μ_0_). The best-fitted curve and full hematocrit–viscosity curve were then represented with respect to *C_dex_* = 0, 15, and 30 mg/mL. Figure 6(Bi) represents the linear regression and full hematocrit–viscosity curve for the test blood (*C_dex_* = 0). Linear regression analysis gave the best-fitted curve as ln (*μ*) = −0.7037 ln (1 − *ϕ*) + 0.1606 (R^2^ = 0.9993); α and *μ*_0_ of the K-D model were identified as *α* = 0.7037 and *μ*_0_ = 1.174 cP. The full hematocrit–viscosity curve was then obtained as $\mu =1.174\;{\left(1\;-\;\phi \right)}^{-0.7037}$. Figure 6(Bii) depicts the linear regression and full hematocrit–viscosity curve for blood (*C_dex_* = 15 mg/mL). Linear regression analysis gave the best-fitted curve as ln (*μ*) = −0.7121 ln (1 − *ϕ*) + 0.7368 (R^2^ = 0.9993). The full hematocrit–viscosity curve was then obtained as $\mu =2.089\;{\left(1\;-\;\phi \right)}^{-0.7121}$. Figure 6(Biii) shows the linear regression and full hematocrit–viscosity curve for blood (*C_dex_* = 30 mg/mL). Linear regression analysis provided the best-fitted curve as ln (*μ*) = −0.6241 ln (1 − *ϕ*) + 1.3091 (R^2^ = 0.9967). The full hematocrit–viscosity curve was then obtained as $\mu =3.703\;{\left(1\;-\;\phi \right)}^{-0.6241}$.

Based on the full hematocrit–viscosity curve obtained from the three datasets, as shown in Figure 6C, variations in *α*, *μ*_0_, and *μ* (*ϕ* = 0.5) are represented as a function of *C_dex_*. The left-side panel shows variations in α with respect to *C_dex_*. The red-dashed line represents the 95% CI. The *α* tended to decrease slightly over *C_dex_*. From the statistical test (i.e., ANOVA-test, *p*-value = 0.432), the dextran solution did not contribute to decreasing *α* significantly. The mid-side panel depicts variations in *μ*_0_ with respect to *C_dex_*. According to the statistical test (i.e., ANOVA-test, *p*-value < 0.001), the dextran solution had a strong impact on *μ*_0_. Considering previous studies [60,61,62], it was confirmed that the dextran solution contributed to increasing *μ*_0_ significantly. Based on the full hematocrit–viscosity curve, blood viscosity was estimated at *ϕ* = 0: *μ* (*ϕ* = 0.5). The right-side panel shows variations in *μ* (*ϕ* = 0.5) with respect to *C_dex_*. The results indicated that blood viscosity was increased significantly at higher concentrations of the dextran solution. The statistical test (i.e., ANOVA-test, *p*-value < 0.001) confirmed that the dextran solution had a strong impact on the viscosity of the suspended blood (*ϕ* = 0.5).

The experimental results show that the exponent (*α*) of the K–D model did not differ significantly with respect to the dextran concentration, whereas *μ*_0_ increased markedly as the dextran concentration increased. These findings suggest that the suspending medium had a strong effect on the *μ*_0_ of the K–D regression model.

### 3.5. Contribution of Rigidified RBCs to Full Hematocrit–Viscosity Curve

In the last subsection, to evaluate the contribution of rigidified RBCs to the full hematocrit–viscosity curve [63,64,65], normal RBCs were rigidified by incubating them into a GA solution, and 1× PBS was selected as the blood medium. Test blood (*ϕ_vol_* = 0.5) was then prepared by adding GA-induced rigidified RBCs into 1× PBS. The blood viscosity of the test blood was measured at a flow rate of 5 mL/h.

**Figure 7:**
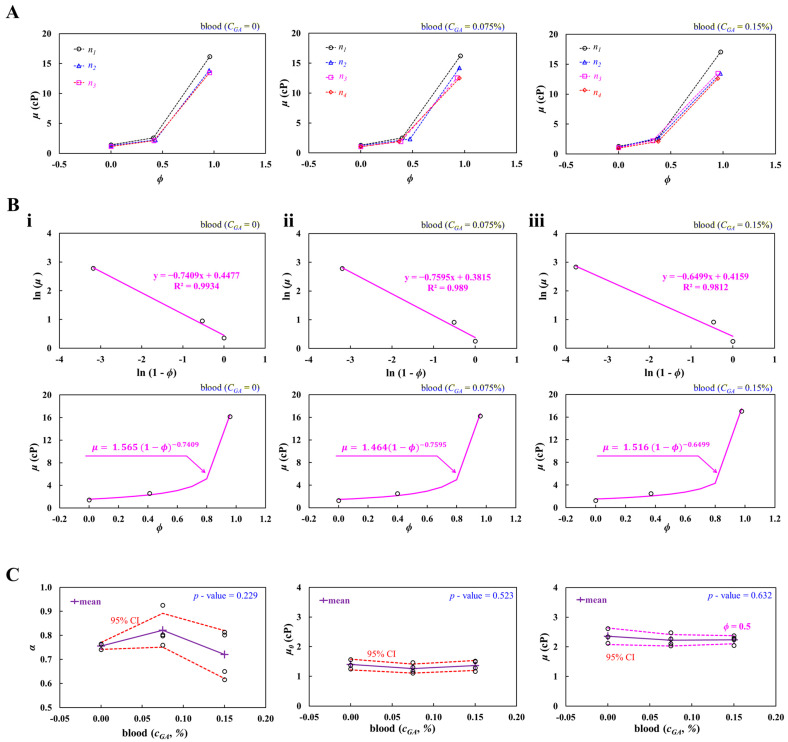
Contribution of RBC deformability to the full hematocrit–viscosity curve. Herein, test blood was prepared by adding GA-induced rigidified RBCs into 1× PBS. The blood viscosity of the test blood was measured at a flow rate of 5 mL/h. (**A**) Three hematocrit–viscosity datasets with respect to GA concentration (*C_GA_* = 0, 0.075%, and 0.15%). (**B**) Full hematocrit–viscosity curve identification with respect to *C_GA_*. (**i**) Linear regression and full hematocrit–viscosity curve for blood (*C_GA_* = 0). (**ii**) Linear regression and full hematocrit–viscosity curve for blood (*C_GA_* = 0.075%). (**iii**) Linear regression and full hematocrit–viscosity curve for blood (*C_GA_* = 0.15%). (**C**) Contribution of GA-treated hardened RBCs to *α*, *μ*_0_, and *μ* (*ϕ* = 0.5). The left-side panel shows variations in α with respect to *C_GA_*. The statistical test (ANOVA-test) gave a *p*-value = 0.229. The mid-side panel depicts variations in *μ*_0_ with respect to *C_GA_*. The ANOVA-test provided a *p*-value = 0.523. The right-side panel shows variations of *μ* (*ϕ* = 0.5) with respect to *C_GA_*. The ANOVA test gave a *p*-value = 0.632.

As shown in Figure 7A, with respect to GA concentration (*C_GA_* = 0, 0.075%, and 0.15%), three hematocrit–viscosity datasets are plotted on the *ϕ*-*μ* axes. Herein, the *C_GA_* denoted 1× PBS. The number of test blood was set to *S_n_* = 3~4. The hematocrit of the test blood (*ϕ*) varied substantially, even though the RBC volume fraction was fixed at 0.5. After GA treatment, RBCs became rigid and tended to adhere to the tube and pipette walls after centrifugation, making it difficult to transfer a precisely defined cell volume. As a result, hematocrit variation increased in hardened blood samples, particularly at *C_GA_* = 0.075% and 0.15%, whereas the control blood showed consistent hematocrit values, confirming reproducibility of the measurement method itself. Based on three hematocrit–viscosity datasets, linear regression analysis was conducted to get the full hematocrit–viscosity curve with respect to *C_GA_*.

Figure (7Bi) shows the linear regression analysis and full hematocrit–viscosity curve for the test blood (*C_GA_* = 0). According to the linear regression analysis, the best-fitted curve was obtained as ln (*μ*) = −0.7409 ln (1 − *ϕ*) + 0.4477 (R^2^ = 0.9934). Two coefficients (*α*, *μ*_0_) of the K-D model were obtained as α = 0.7409 and *μ*_0_ = 1.564 cP. The full hematocrit–viscosity curve was then obtained as $\mu =1.565{\left(1-\phi \right)}^{-0.7409}$. Figure 7(Bii) depicts the linear regression analysis and full hematocrit–viscosity curve for blood (*C_GA_* = 0.075%). The linear regression analysis gave the best-fitted curve as ln (*μ*) = −0.7595 ln (1 − *ϕ*) + 0.3815 (R^2^ = 0.989). Two coefficients (*α*, *μ*_0_) of the K-D model were obtained as α = 0.7595 and *μ*_0_ = 1.464 cP. The full hematocrit–viscosity curve was then obtained as $\mu =1.464{\left(1-\phi \right)}^{-0.7595}$. Figure 7(Biii) exhibits the linear regression analysis and full hematocrit–viscosity curve for blood (*C_GA_* = 0.15%). The linear regression analysis gave the best-fitted curve as ln (*μ*) = −0.6499 ln (1 − *ϕ*) + 0.4159 (R^2^ = 0.9812). The full hematocrit–viscosity curve was then obtained as $\mu =1.516{\left(1-\phi \right)}^{-0.6499}$.

As shown in Figure 7C, variation in *α*, *μ*_0_, and *μ* (*ϕ* = 0.5) are plotted as a function of *C_GA_*. The left-side panel shows variations in *α* with respect to *C_GA_*. The red-dashed line represents the 95% CI. From the statistical test (i.e., ANOVA-test, *p*-value = 0.229), the GA-treated hardened RBCs did not contribute to decreasing *α* significantly. Specifically, the *α* decreased slightly between *C_GA_* = 0.075% and *C_GA_* = 0.15%. The mid-side panel depicts variations in *μ*_0_ with respect to *C_GA_*. According to the statistical test (i.e., ANOVA-test, *p*-value = 0.523), the GA-induced rigidified RBCs did not affect *μ*_0_ significantly. Furthermore, because 1× PBS was used as the suspending medium for the test blood, *μ*_0_ was confirmed to be independent of the GA solution concentration. The right-side panel shows variations in *μ* (*ϕ* = 0.5) with respect to *C_GA_*. The blood viscosity did not show a substantial difference with respect to *C_GA_*. The statistical test (i.e., ANOVA-test, *p*-value = 0.632) confirmed that the rigidified RBCs did not have a strong impact on the viscosity of suspended blood (*ϕ* = 0.5). According to the previous study [61], blood viscosity (*ϕ_vol_* = 0.3) did not show a substantial difference up to *C_G_*_A_ = 0.1%. On the other hand, RBC deformability decreases progressively with increasing GA concentration, including a nearly linear decline of up to 0.08% GA by ektacytometry and marked rigidification even at low GA levels such as 0.01~0.025% [66]. However, the present method did not exhibit a substantial difference in blood viscosity within 0.15% of the GA solution. Thus, it was inferred that the present results showed consistent trends with respect to *C_GA_*.

From the experimental results, the rigidified RBCs did not have a strong impact on the coefficients of the K-D regression model. Blood viscosity (*ϕ* = 0.5) remained constant up to *C_GA_* = 0.15%.

As a limitation of this study, the proposed method was validated only under limited experimental conditions, including specific flow rates, dextran concentrations, and GA concentrations. Because the experiments were conducted using in vitro test blood prepared with a dextran solution or GA-induced rigidified RBCs in 1× PBS, the results may not fully represent the rheological behavior of native whole blood under physiological conditions. Therefore, further studies with larger sample sizes and broader experimental conditions will be needed to validate the robustness and practical applicability of the proposed method. In addition, to measure blood viscosity resulting from RBC aggregation in a microfluidic environment, the flow rate should be set to less than 0.15 mL/h (i.e., shear rate < 100 s^−1^). However, as the current microfluidic system did not guarantee a consistent flow rate at the low shear rate, blood viscosity was measured at a sufficiently high shear rate. It will be necessary to improve the blood delivery system, which can maintain a low flow rate, and measure blood viscosity consistently.

## 4. Conclusions

This study demonstrated the feasibility of reconstructing the full hematocrit–viscosity curve from only three selected datasets using the K–D regression model. The results suggested that the full hematocrit–viscosity curve obtained from the three selected datasets was in good agreement with the experimental data and yielded a lower fitting error than the conventional method using all datasets. The exponent of the K–D model was strongly influenced by the midpoint hematocrit, whereas *μ*_0_ was mainly affected by the suspending medium. In contrast, GA-induced rigidified RBCs did not significantly affect *μ*_0_ within a 0.15% concentration. In conclusion, the proposed method provided a simple, efficient, and reliable approach for estimating the full hematocrit–viscosity curve.

## Data Availability

The original contributions presented in this study are included in the article material. Further inquiries can be directed to the corresponding author.
